# Generation of an induced pluripotent stem cell line (TRNDi012-B) from Fibrodysplasia Ossificans Progressiva (FOP) patient carrying a heterozygous mutation c. 617G > A in the *ACVR1* gene

**DOI:** 10.1016/j.scr.2021.102424

**Published:** 2021-06-07

**Authors:** Xiuli Huang, Amanda Roeder, Rong Li, Jeanette Beers, Chengyu Liu, Jizhong Zou, Paul B. Yu, Wei Zheng

**Affiliations:** a National Center for Advancing Translational Sciences, National Institutes of Health, Bethesda, MD, USA; b iPSC Core, National Heart, Lung and Blood Institute, National Institutes of Health, Bethesda, MD, USA; c Transgenic Core, National Heart, Lung and Blood Institute, National Institutes of Health, Bethesda, MD, USA; d Brigham and Women’s Hospital and Harvard Medical School, Division of Cardiovascular Medicine, Boston, MA, USA

## Abstract

Fibrodysplasia ossificans progressiva (FOP) is a rare autosomal dominant disorder of progressive ossification of skeletal muscle, fascia, tendons, and ligaments. Most FOP cases are caused by a heterozygous c. 617G > A mutation in the *ACVR1* gene which encodes a gain-of-function of bone morphogenetic protein type I receptor. A human induced pluripotent stem cell (iPSC) line was generated from the dermal skin fibroblasts of a FOP patient who carries the c. 617G > A mutation in the *ACVR1* gene. This iPSC line provides an attractive resource for FOP disease modeling.

## 1. Resource utility

This human induced pluripotent stem cell line is a useful tool for establishing *in vitro* disease model and for applications in drug development to treat FOP.

## 2. Resource details

Fibrodysplasia ossificans progressiva (FOP) is a rare genetic disease characterized by congenital malfunctions of great toes and progressive heterotopic ossification of soft tissues, that results in severe restriction of joint function and premature mortality (Kaplan et al., 2009). The majority of FOP patients carry a heterozygous missense mutation (c.617G > A; p.R206H) in the *ACVR1* gene, which encodes activin A type I receptor /activin receptor-like kinase 2 (ACVR1/ALK2), a morphogenetic protein (BMP) type I receptor (Shore et al., 2006). The mutation causes a gain-of-function of activation of ACVR1, leading to the transformation of connective tissue and muscle tissue into a secondary skeleton (Hatsell et al., 2015). FOP has a prevalence of approximately 1 in 2 million people worldwide (Baujat et al., 2017). Currently, no approved treatment exists for FOP patients.

**Fig. 1.**
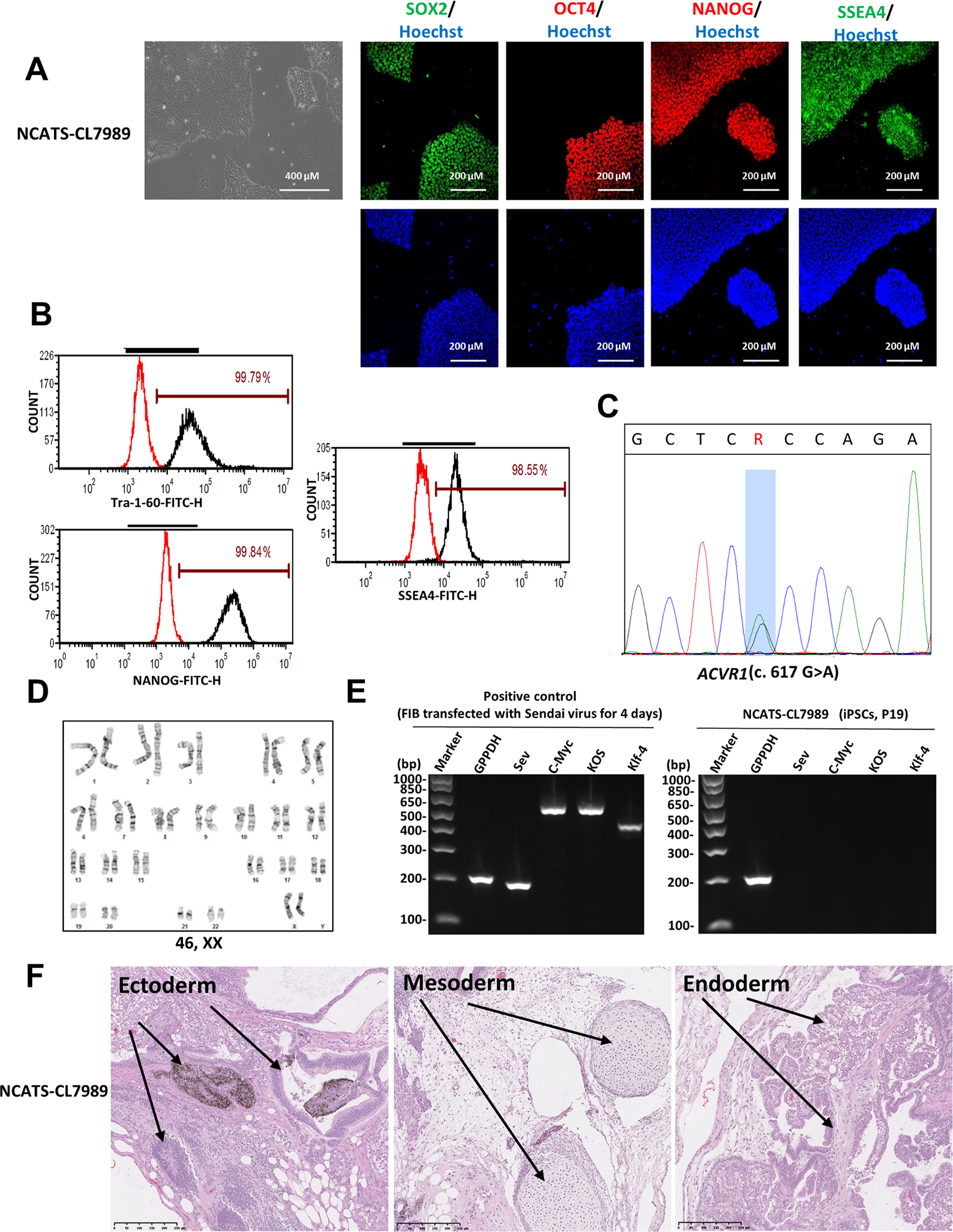
Characterization of NCATS-CL7989 iPSC line. (A) Left: Phase contrast image of NCATS-CL7989 colonies. Right: Immunofluorescence images of iPSCs positive for stem cell markers: SOX2, OCT4, NANOG and SSEA4. Nucleus is labelled with Hoechst 33342 (blue). (B) Flow cytometry analysis of pluripotency protein markers: TRA-1–60, NANOG and SSEA4. (C) Detection of heterozygous gene mutation of c.617G > A in the *ACVR1* gene. (D) Cytogenetic analysis showing a normal karyotype (46, XX). (E) RT-PCR confirmation for the clearance of the Sendai virus from reprogrammed cells. Sendai virus vector transduced fibroblasts was used as a positive control. (F) Pathological analysis of teratoma from NCATS-CL7989 iPSC showing a normal ectodermal, mesodermal and endodermal differentiation.

In this study, we generated a human iPSC line (TRNDi012-B, also named as NCATS-CL7989) from patient skin fibroblast (GM00513) which was isolated from a 16-year-old female FOP patient carrying a heterozygous mutation (c.617G > A; p.R206H) in ACVR1 gene. (see Table 1). A non-integrating CytoTune-Sendai viral vector kit (A16517, ThemoFisher Scientific) containing OCT3/4, KLF4, SOX2 and C-MYC pluripotency transcription factors was employed to transduce the patient cells using a previously described method (Beers et al., 2015). A single iPSC colony, termed NCATS-CL7989, was isolated and characterized. Fig. 1A show that the iPS cells displayed a standard pluripotent stem cell morphology under phase contrast microscopy and expressed pluripotent markers SOX2, OCT4, NANOG and SSEA4 in the immunocytochemistry assay. In addition, flow cytometric analysis showed that the expression levels of NANOG, SSEA4 and cell surface marker TRA-1-60 were over 98% (Fig. 1B). The mutation (c.617G > A) of the *ACVR1* gene in the NCATS-CL7989 iPSC line was confirmed by Sanger sequencing the PCR product harboring the single nucleotide variation (SNV) (Fig. 1C). At passage 7, cells showed a normal karyotype (46, XX), as confirmed by the G-banded karyotyping (Fig. 1D). The Sendai virus vector (Sev) clearance was verified with reverse transcription polymerase chain reaction (RT-PCR) using Sev-specific primers. The Sev vector disappeared at passage 19 (Fig. 1E). The iPSCs was not contaminated with mycoplasma (Supplementary Fig. S1), and 16 loci from NCATS-CL7989 iPS cells and GM00513 FOP fibroblasts determined by the short tandem repeat (STR) DNA profile were identical (information available from the authors). Furthermore, the pluripotency of this iPSC line was confirmed by a teratoma formation experiment that exhibited its ability to differentiate into all three germ layers (Ectoderm, Mesoderm and Endoderm) *in vivo* (Fig. 1F).

## 3. Materials and methods

### 3.1. Cell culture

The FOP patient fibroblasts (GM00513, Coriell Cell Repository) were maintained in DMEM containing 15% fetal bovine serum (HyClone). NCATS-CL7989 iPSCs were cultured in StemFlex™ medium (Thermo Fisher Scientific) on Matrigel (Corning, Cat# 354277)-coated plates at 37 °C in humidified air with 5% CO_2_. The cells were passaged with 0.5 mM Ethylenediaminetetraacetic acid (EDTA) at 1:6 ratio when they reached 80% confluency.

### 3.2. Reprogramming of human skin fibroblasts

Using a CytoTune™-iPS 2.0 Sendai Reprogramming Kit (A16517, ThermoFisher Scientific) containing c-MYC, KLF4, OCT3/4, and SOX2 pluripotency transcription factors, the FOP patient fibroblasts (GM00513) were reprogrammed to iPSCs as previously reported (Beers et al., 2015).

### 3.3. Mutation analysis

Genomic mutation analysis was contracted to Codex BioSolutions (Gaithersburg, MD, USA). Briefly, genomic DNA was extracted from the cell pellet of FOP iPSCs with a PureLink™ Genomic DNA Mini kit (K182002, ThermoFisher Scientific). The mutation in the *ACVR1* gene was analyzed by a DNA sequencing of PCR amplified product. With the specific primers listed in Table 2 and JumpStart Taq ReadyMix (P2893, Sigma-Aldrich), 40 cycles of PCR were performed with annealing temperature at 56 °C and extension time of 70 s. Sanger sequencing analysis was then employed for genotyping the heterozygous mutation, resulting in confirmation of the mutation of c. 617G > A in the *ACVR1* gene.

### 3.4. Immunocytochemistry staining

The iPSCs in a 96-well plate were fixed in 4% paraformaldehyde for 15 min followed by a plate wash with Dulbecco’s phosphate-buffered saline (DPBS) and cell permeabilization with 0.1% Triton X-100 in DPBS for 15 min. The Image-iT™ FX Signal Enhancer (I36933, ThermoFisher Scientific) was added to the fixed cells and incubated for 40 min at room temperature. The primary antibodies against SOX2, OCT4, NANOG and SSEA4 diluted in the Image-iT™ FX Signal Enhancer were then added and incubated at 4 °C overnight. After washing with DPBS, a corresponding secondary antibody conjugated with Alexa Fluor 488 or Alex Fluor 594 as these listed in Table 2 was added to the cells for 1 h incubation at room temperature. After the cells were washed and stained with Hoechst 33342 for 15 min, an INCell Analyzer 2200 imaging system (GE Healthcare) was used imaging analysis with 20X objective lens and Texas Red, FITC and DAPI filter sets.

### 3.5. Flow cytometry analysis

The iPSCs were harvested using TrypLE Express enzyme (12605010, ThermoFisher Scientific) and fixed with 4% paraformaldehyde for 10 min at room temperature. After the cells were washed with DPBS and permeabilized with 0.2% Tween-20 in DPBS for 10 min at room temperature, fluorophore conjugated antibodies (Table 2) were incubated with the cells for 1 h at 4 °C on a shaker. A BD AccuriC6 Flow Cytometer (BD Biosciences) was used for the flow cytometry analysis.

### 3.6. G-banded karyotyping

The G-banding karyotype analysis of the iPS cells at the passage-7 was performed at the WiCell Research Institute (Madison, WI) using a standard cytogenetic protocol. Cells on a slide were incubated with ethidium bromide and colcemid, followed by a fixation in hypotonic solution. Leishman’s stain was then used to stain the metaphase cells. A total of 20 randomly selected metaphases were analyzed.

### 3.7. Short tandem repeat (STR) DNA profile analysis

The Short Tandem Repeat (STR) analysis of original patient fibroblasts and derived iPSCs was carried out by the Translational Research Initiatives in Pathology (TRIP) Laboratory at University of Wisconsin–Madison. A PowerPlex® 16 HS System (DC2101, Promega) was employed in multiplex PCR to amplify fifteen STR loci (D5S818, D13S317, D7S820, D16S539, vWA, TH01, TPOX, CSF1PO, D18S51, D21S11, D3S1358, D8S1179, FGA, Penta D, Penta E) and Amelogenin (AMEL, a gender determining marker). Capillary electrophoresis of the PCR products was carried out on an ABI 3500xL Genetic Analyzer (Applied Biosystems) using the Internal Lane Standard 600 (ILS 600) (Promega). A GeneMapper® v 4.1 software (Applied Biosystems) was used for data analysis.

### 3.8. Mycoplasma detection

Using a MycoAlert^™^ Mycoplasma Detection Kit (LT07-218, Lonza), mycoplasma detection was performed following the manufacturer’s instruction. Ratio B/A < 0.9 indicates mycoplasma negative, Ratio between 0.9 and 1.2 indicates mycoplasma ambiguous results, and Ratio B/A > 1.2 indicates mycoplasma positive.

### 3.9. Testing for Sendai reprogramming vector clearance

Total RNA was isolated from the iPSCs (NCATS-CL7989) at passage 19 using a RNeasy Plus Mini Kit (74034, Qiagen). The total RNA from human fibroblasts (Coriell Institute, GM05659) transduced with Sendai virus for 4 days was used as a positive control. A SuperScript™ III First-Strand Synthesis SuperMix (18080400, ThermoFisher Scientific) was used for the cDNA synthesis from 1 μg RNA and random hexamers, followed by a PCR (primers listed in Table 2) performed using a Platinum II Hot-Start PCR Master Mix (14000012, ThermoFisher Scientific) with the following program: 94 °C, 2 mins; 30 cycles of (94 °C, 15 s, 60 °C, 15 s and 68 °C, 15 s). The PCR products were loaded to an E-Gel® 1.2% with SYBR Safe™ gel, ran at 120 V electric field, and imaged by G: Box Chemi-XX6 gel doc system (Syngene, Frederick, MD).

### 3.10. Teratoma formation assay

After the patient iPSCs (NCATS-CL7989) were dissociated with DPBS containing 0.5 mM EDTA, the cells (approximately 1 × 10^7^) were resuspended in 400 μl culture medium supplied with 25 mM HEPES pH 7.4. The cells, stored on ice prior to injection, were mixed with 200 μl of cold Matrigel (354277, Corning) and injected subcutaneously into NSG mice (JAX No. 005557) at 150 μl per injection site. Visible tumors were removed after 6–8 weeks that were fixed in 10% Neutral Buffered Formalin, and then embedded in paraffin for staining with hematoxylin and eosin.

## Supplementary Material

Supplemental Fig. S1

## Figures and Tables

**Table 1:** Characterization and Validation.

Classification	Test	Result	Data

Morphology	Photography	Normal	Fig. 1 Panel A
Phenotype	Immunocytochemistry	SOX2, OCT4, NANOG, SSEA-4	Fig. 1 Panel A
	Flow cytometry	TRA-1-60 (99.79%)	Fig. 1 Panel B
		NANOG (99.84%)	
		SSEA4 (98.55%)	
Genotype	Karyotype (G-banding) and resolution	46,XX Resolution: 425–500	Fig. 1 Panel D
Identity	Microsatellite PCR (mPCR) OR	Not performed	N/A
	STR analysis	16 sites tested; all sites matched	Available from the authors
Mutation analysis (IF APPLICABLE)	Sequencing	*ACVR1*, c.617G > A	Fig. 1 Panel C
	Southern Blot OR WGS	N/A	N/A
Microbiology and virology	Mycoplasma	Mycoplasma testing by luminescence.	Supplementary Fig. S1
		Negative	
Differentiation potential	Teratoma formation	Teratoma with three germ layers: Ectoderm, Mesoderm and Endoderm	Fig. 1 Panel F
Donor screening (OPTIONAL)	HIV 1 + 2 Hepatitis B, Hepatitis C	N/A	N/A
Genotype additional info (OPTIONAL)	Blood group genotyping	N/A	N/A
	HLA tissue typing	N/A	N/A

**Table 2:** Reagents details.

Antibodies used for immunocytochemistry/flow-cytometry			
	Antibody	Dilution	Company Cat # and RRID

Pluripotency Markers	Mouse anti-SOX2	1:50	R & D systems, Cat# MAB2018, RRID: AB_358009
Pluripotency Markers	Rabbit anti-NANOG	1:400	Cell signaling, Cat# 4903, RRID: AB_10559205
Pluripotency Markers	Rabbit anti-OCT4	1:400	Thermo Fisher, Cat# A13998, RRID: AB_2534182
Pluripotency Markers	Mouse anti-SSEA4	1:1000	Cell signaling, Cat# 4755, RRID: AB_1264259
Secondary Antibodies	Donkey anti-Mouse IgG (Alexa Fluor 488)	1:400	Thermo Fisher, Cat# A21202, RRID: AB_141607
Secondary Antibodies	Donkey anti-Rabbit IgG (Alexa Fluor 594)	1:400	Thermo Fisher, Cat# A21207, RRID: AB_141637
Flow Cytometry Antibodies	Anti-Tra-1-60-DyLight 488	1:50	Thermo Fisher, Cat# MA1-023-D488X, RRID: AB_2536700
Flow Cytometry Antibodies	Anti-Nanog-Alexa Fluor 488	1:50	Millipore, Cat# FCABS352A4, RRID: AB_10807973
Flow Cytometry Antibodies	Anti-SSEA-4-Alexa Fluor 488	1:50	Thermo Fisher, Cat# 53-8843-41, RRID: AB_10597752
Flow Cytometry Antibodies	Mouse-IgM-DyLight 488	1:50	Thermo Fisher, Cat# MA1-194-D488, RRID: AB_2536969
Flow Cytometry Antibodies	Rabbit IgG-Alexa Fluor 488	1:50	Cell Signaling, Cat# 4340S, RRID: AB_10694568
Flow Cytometry Antibodies	Mouse IgG3-FITC	1:50	Thermo Fisher, Cat# 11-4742-42, RRID: AB_2043894
**Primers**			

	**Target**	**Forward/Reverse primer (5′-3′)**	

Sev specific primers (RT-PCR)	Sev/181 bp	GGA TCA CTA GGT GAT ATC GAG C/ ACC AGA CAA GAG TTT AAG AGA TAT GTA TC	
Sev specific primers (RT-PCR)	KOS/528 bp	ATG CAC CGC TAC GAC GTG AGC GC/ ACC TTG ACA ATC CTG ATG TGG	
Sev specific primers (RT-PCR)	Klf4/410 bp	TTC CTG CAT GCC AGA GGA GCC C/ AAT GTA TCG AAG GTG CTC AA	
Sev specific primers (RT-PCR)	C-Myc/523 bp	TAA CTG ACT AGC AGG CTT GTC G/ TCC ACA TAC AGT CCT GGA TGA TGA TG	
House-Keeping gene (RT-PCR)	GAPDH/197 bp	GGA GCG AGA TCC CTC CAA AAT/ GGC TGT TGT CAT ACT TCT CAT GG	
Targeted mutation analysis (PCR)	ACVR1 (c. 617G > A)/350 bp	CCA GTC CTT CTT CCT TCT TCC/ AGC AGA TTT TCC AAG TTC CAT C	

**Resource Table:** 

Unique stem cell line identifier	TRNDi012-B
Alternative name(s) of stem cell line	NCATS-CL7989 or HT216B
Institution	National Institutes of Health National Center for Advancing Translational Sciences Bethesda, Maryland, USA
Contact information of distributor	Dr. Wei Zheng Wei.Zheng@nih.gov
Type of cell line	iPSC
Origin	Human
Additional origin info	Age: 16-year-old Sex: Female Ethnicity: Caucasian
Cell Source	Dermal fibroblasts
Clonality	Clonal
Method of reprogramming	Integration-free Sendai viral vectors containing OCT3/4, KLF4, SOX2, and c-MYC pluripotency transcription factors
Genetic Modification	Yes
Type of Modification	Hereditary
Associated disease	Fibrodysplasia ossificans progressiva (FOP)
Gene/locus	*ACVR1*, chromosomal location:2q23-24, c.617G > A; p. R206H
Method of modification	N/A
Name of transgene or resistance	N/A
Inducible/constitutive system	N/A
Date archived/stock date	2015
Cell line repository/bank	https://hpscreg.eu/cell-line/TRNDi012-B
Ethical approval	NIGMS Informed Consent Form was obtained from the patient at time of sample submission. Confidentiality Certificate: CC-GM-15-004
