# Supplementary figures and images for: Generation of an induced pluripotent stem cell line (TRNDi012-B) from Fibrodysplasia Ossificans Progressiva (FOP) patient carrying a heterozygous mutation c. 617G > A in the ACVR1 gene
Source: Stem Cell Res. Author manuscript; Available in PMC 2022 Aug 31. (PMC9428929; doi:10.1016/j.scr.2021.102424)

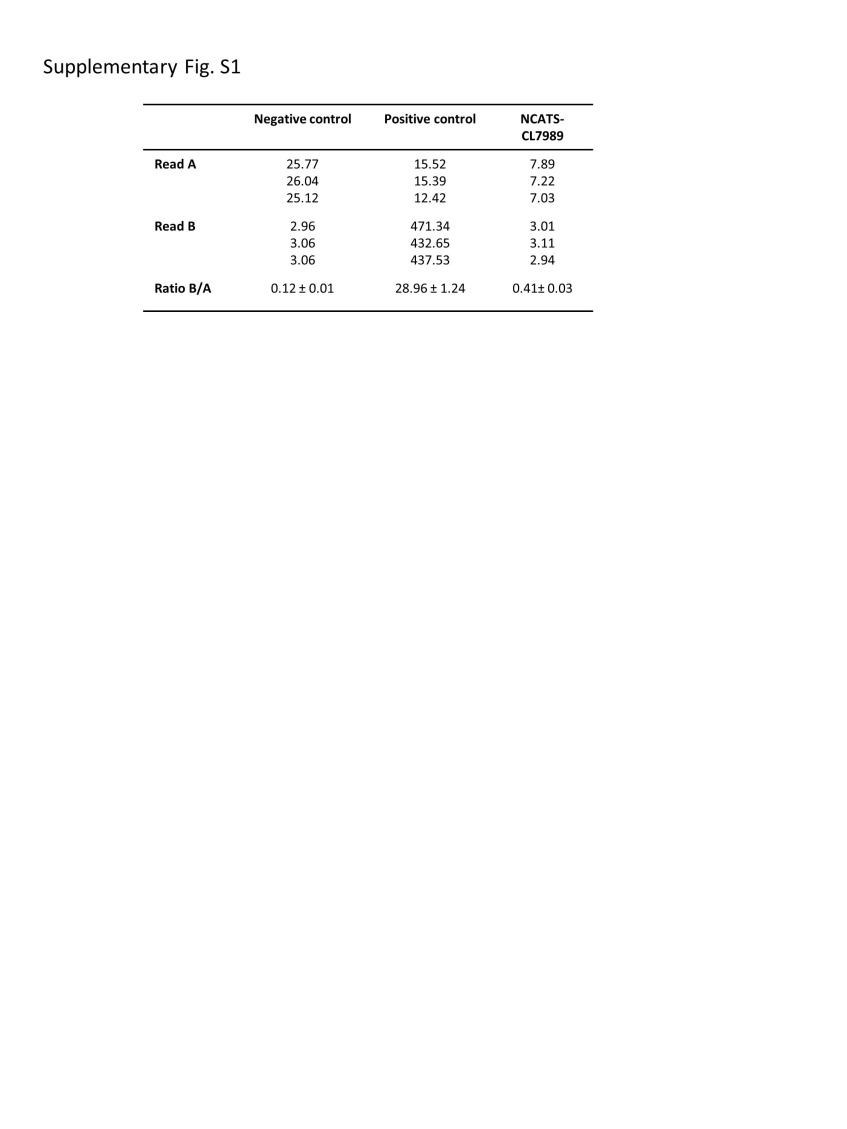

Supplement: Supplemental Fig. S1 [file NIHMS1820176-supplement-Supplemental_Fig__S1.jpg]
